# Post-flexible Bronchoscopy Escherichia coli Empyema: A Rare Complication or an Inflammatory Bacterial Translocation?

**DOI:** 10.7759/cureus.96852

**Published:** 2025-11-14

**Authors:** Ukasha Moazzam, Bakht Noor Khurshid, Cyrus Daneshvar

**Affiliations:** 1 Geriatrics, Derriford Hospital/ University Hospitals Plymouth NHS Trust, Plymouth, GBR; 2 Acute Medicine, Derriford Hospital/ University Hospitals Plymouth NHS Trust, Plymouth, GBR; 3 Respiratory Medicine, Derriford Hospital/ University Hospital Plymouth NHS Trust, Plymouth, GBR

**Keywords:** airway colonisation, bacterial translocation, bronchoceles, bronchoscopy complication, empyema, escherichia coli

## Abstract

Empyema is an unusual complication of diagnostic bronchoscopy. We report a case of a male patient in his late 70s with bronchiectasis who developed a right lower lobe empyema 10 days after undergoing flexible bronchoscopy for incidentally detected lung nodules. Bronchial washings grew *Staphylococcus aureus* (*S. aureus*) and the atypical Gram-negative bacillus *Pantoea septica *(*P. septica*), while subsequent pleural fluid culture yielded *Escherichia coli *(*E. coli)*. This case highlights the risk of post-bronchoscopy infectious complications in patients with structural lung disease and illustrates how discordance between airway and pleural isolates can complicate antimicrobial decision-making.

## Introduction

Flexible bronchoscopy with bronchoalveolar lavage (BAL) is widely used for both diagnosis and sampling of the lower respiratory tract [1]. Indications include assessment of airway obstruction, suspected malignancy, unexplained hemoptysis, chronic infection, and obtaining material for microbiological or cytological analysis [1].

While the procedure is generally safe, recognized complications include bleeding, pneumothorax, and, less commonly, infection [2]. Empyema, defined as pus within the pleural space, typically follows pneumonia; its occurrence after bronchoscopy is rare [2, 3]. Fewer than 15 cases of post-bronchoscopy lung abscess or empyema were reported between 1981 and 2020 [3].

Patients with structural lung disease such as bronchiectasis or bronchoceles are particularly vulnerable, being prone to colonization with atypical or opportunistic pathogens [4]. This may increase susceptibility to progression from colonization to active infection following airway instrumentation.

## Case presentation

**Figure 1:**
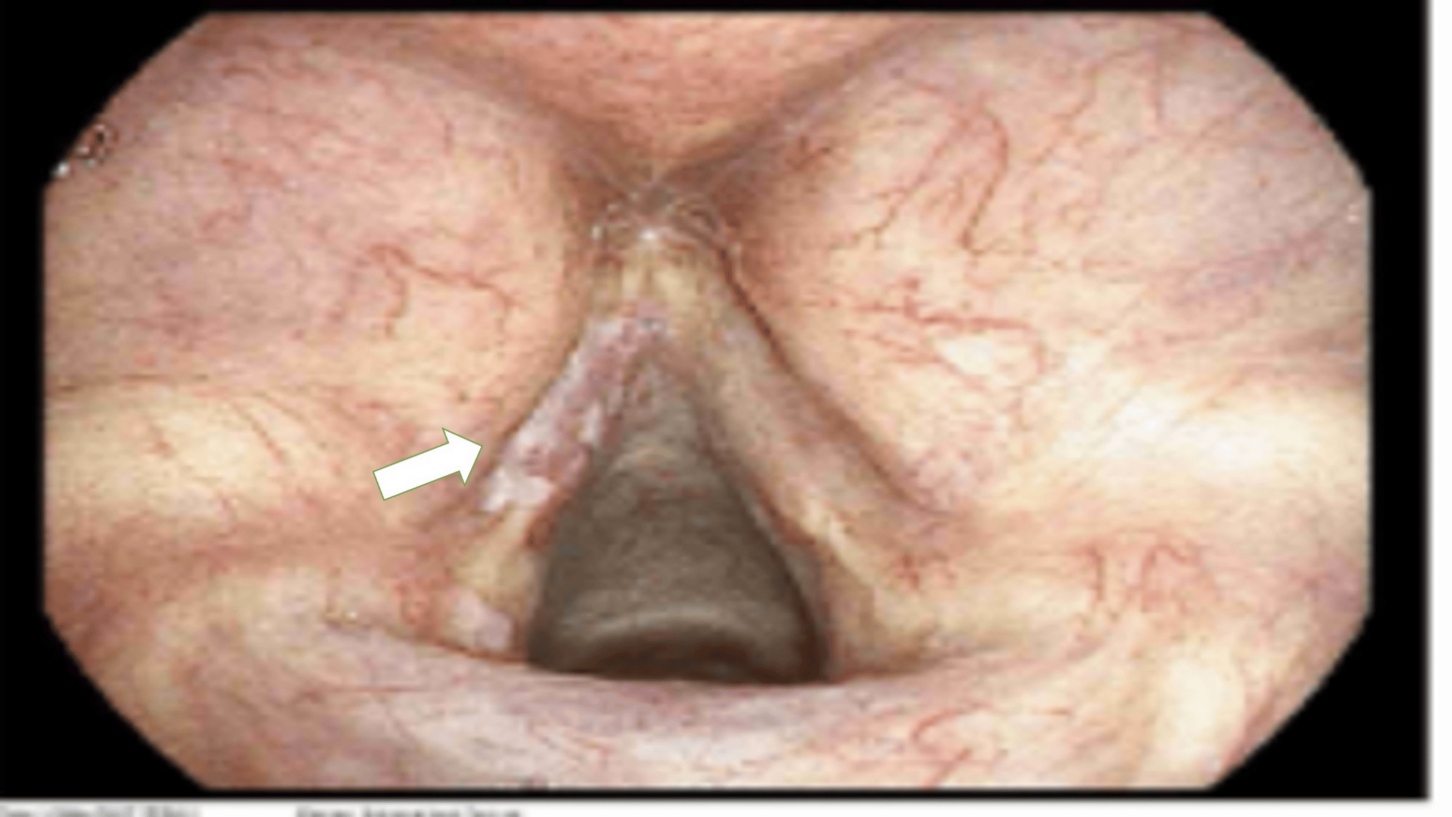
Bronchoscopic view of the vocal cords. The left side had a white plaque and was excoriated (indicated by the white arrow).

A 76-year-old man with asthma-chronic obstructive pulmonary disease (COPD) overlap, bronchiectasis, hypertension, a history of smoking, and a pancreatic cyst under surveillance underwent routine magnetic resonance cholangiopancreatography (MRCP) in December 2024, which incidentally showed bilateral lower-lobe nodules. Computed tomography (CT) of the thorax suggested bronchoceles. He was independent in activities of daily living and had no recent respiratory infections. An elective flexible bronchoscopy was performed as a day-case procedure on 10 March 2025 for diagnostic evaluation of probable right lower-lobe and lingular bronchoceles. This was undertaken to exclude infection and to obtain lavage samples before potential further intervention. The procedure revealed left vocal cord leukoplakia (Figure 1), narrowed and inflamed right lower lobe airways (medial basal segment), and no endobronchial tumour. No biopsies were taken, but bronchial washings were obtained from the right lower lobe. He was discharged the same day with no immediate complications.

Over the next 10 days, he developed progressive breathlessness, a productive cough, and malaise. On 21^st^ March 2025, he presented to the emergency department hypoxic, tachypnoeic, and pyrexial, with pleuritic right-sided chest pain. Bilateral basal crackles on examination and a new oxygen requirement were noted.

**Figure 2:**
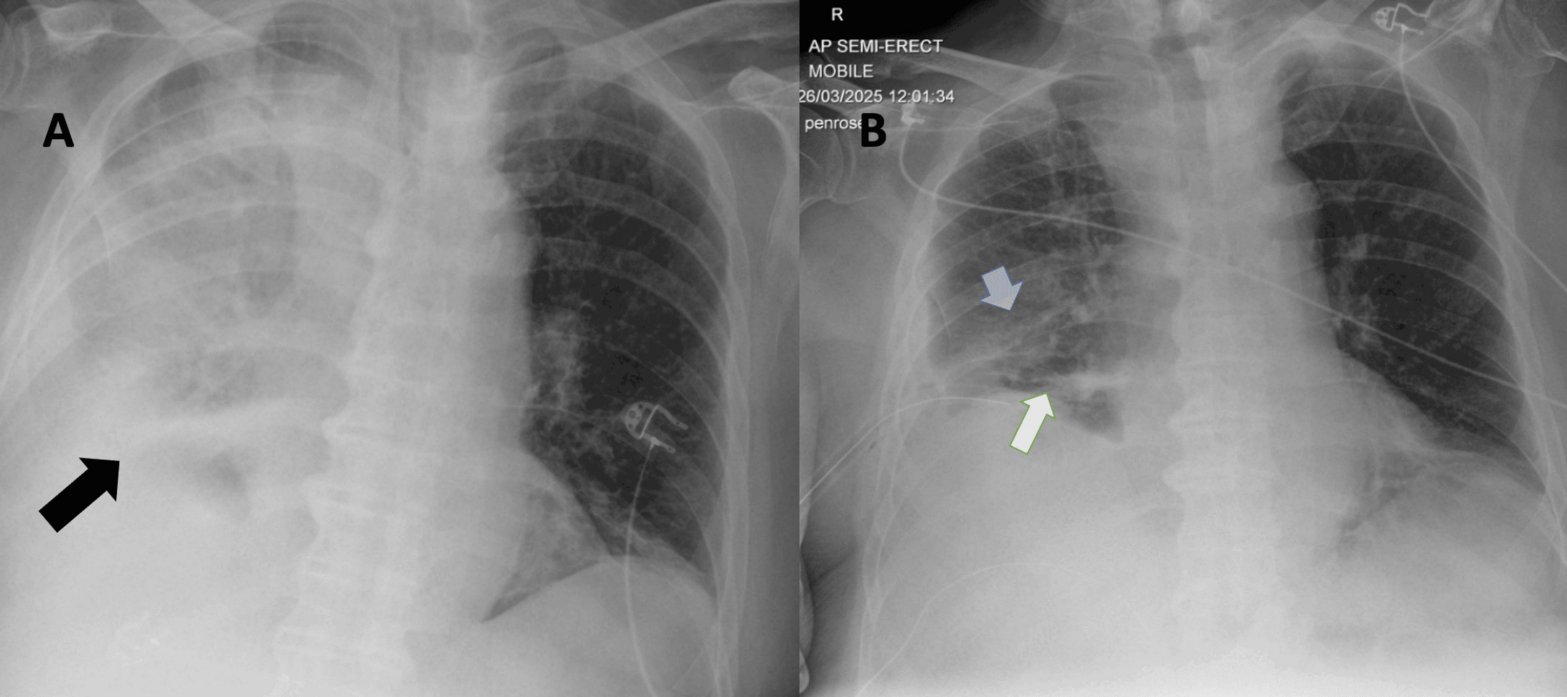
Serial chest radiographs (anteroposterior, semi-erect) (A) 25^th^ March, 2025: Extensive right-sided opacification consistent with consolidation and associated pleural effusion (black arrow); (B) 26^th^ March, 2025: Post chest-drain insertion with one drain tip projected over the right mid-zone (blue arrow) and another within the cardiophrenic recess (white arrow); interval improvement in right-sided aeration.

The initial chest radiograph (21^st^ March) showed a clear right haemithorax without consolidation or effusion. In contrast, a repeat film (25^th^ March) demonstrated new extensive right-sided consolidation with an associated pleural effusion (Figure 2A), prompting further evaluation with thoracic ultrasound (TUS) and CT scan.

**Figure 3:**
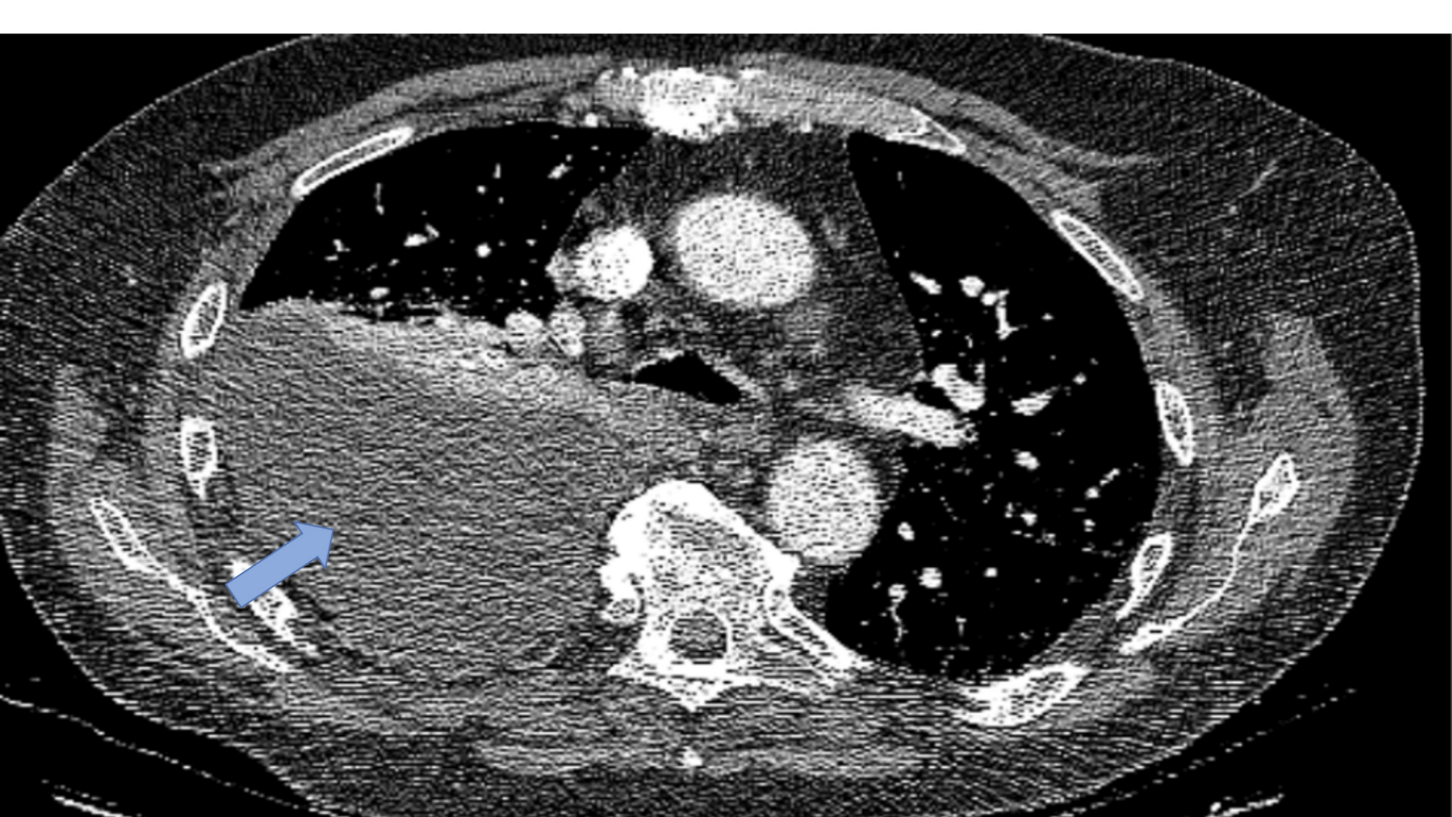
CT scan of the chest (axial plane, 25th March, 2025). The scan demonstrates a marked increase in the size of the right pleural effusion (blue arrow) compared with the scan performed four days earlier. There is a progressive and complete collapse of the right upper and middle lobes. Central fluid-filled cystic areas within the right lower lobe are less demonstrated due to surrounding consolidation/collapse.

A contrast-enhanced CT of the thorax (25^th^ March) confirmed a right lower-lobe intrapulmonary abscess with a large, loculated pleural effusion (Figure 3). TUS, performed on the same day, showed a complex, heavily septated, echogenic pleural effusion, with a maximum depth of approximately 9 cm over two rib spaces. A 12 F Seldinger intercostal drain was inserted under direct ultrasound guidance, draining approximately 400 mL of cloudy fluid; samples were sent for microbiological analysis.

He was admitted to the intensive care unit (ICU) for respiratory failure and commenced on high-flow nasal oxygen and empirical broad-spectrum piperacillin-tazobactam. Bronchial washings grew *Staphylococcus aureus* (*S. aureus*) and *Pantoea septica* (*P. septica*) (Figure 2B). In view of the isolation of S. aureus and the clinical severity, possible Panton-Valentine leukocidin (PVL)-producing *S. aureus* was considered. Because the isolates had already been discarded and PVL testing could not be performed, antibiotic cover was changed to a β-lactam (co-amoxiclav) and clindamycin for toxin suppression. When the pleural aspirate later yielded *Escherichia coli* (*E. coli*) sensitive to meropenem, therapy was escalated accordingly.

He developed new-onset atrial fibrillation, which responded to amiodarone, and transthoracic echocardiography showed no evidence of endocarditis (Table 1).

**Table 1:** Summary of microbiological, cardiac, and imaging findings

Domain	Findings
Microbiology	Bronchial washings: *Staphylococcus aureus* (*S. aureus)* and *Pantoea septica (P. septica)*. Sensitivities: *S. aureus* – flucloxacillin, erythromycin, doxycycline. *P. septica* – meropenem, levofloxacin (resistant to amoxicillin, cefalexin). Pleural fluid: *Escherichia coli (E. coli; *sensitive to meropenem). Additional tests: *Aspergillus precipitins*, mycobacterial PCR/culture, and HIV serology – all negative.
Cardiac evaluation	Transthoracic echocardiogram (26 March 2025): no vegetations.
Imaging summary	21 March 2025: CT abdomen/pelvis showed right lower-lobe (RLL) infective changes and abscess. 25 March 2025: Chest drain insertion – drained a moderate amount of seropurulent fluid. 25 March 2025: Thoracic ultrasound – complex, septated effusion (9 cm depth). 25 June 2025: CT of the thorax – improved empyema, RLL bronchoceles, multinodular goitre, lingular bronchiectasis. 7 July 2025: Thoracic ultrasound – small residual posterior collection (about 2.8 cm, not aspirated, patient clinically improved). 24 July 2025: CT pulmonary angiography – no pulmonary embolism, stable residual empyema.

Over the following two weeks, his respiratory status and inflammatory markers steadily improved with ongoing drainage and targeted antimicrobial therapy. Serial chest radiographs demonstrated progressive resolution of the right-sided consolidation and effusion (Figure 2B). Drain output diminished, and the intercostal drain was removed on 9^th^ April, 2025. Once clinically stable, he was stepped down from the ICU and discharged on oral levofloxacin and metronidazole to complete a six-week antibiotic course.

**Figure 4:**
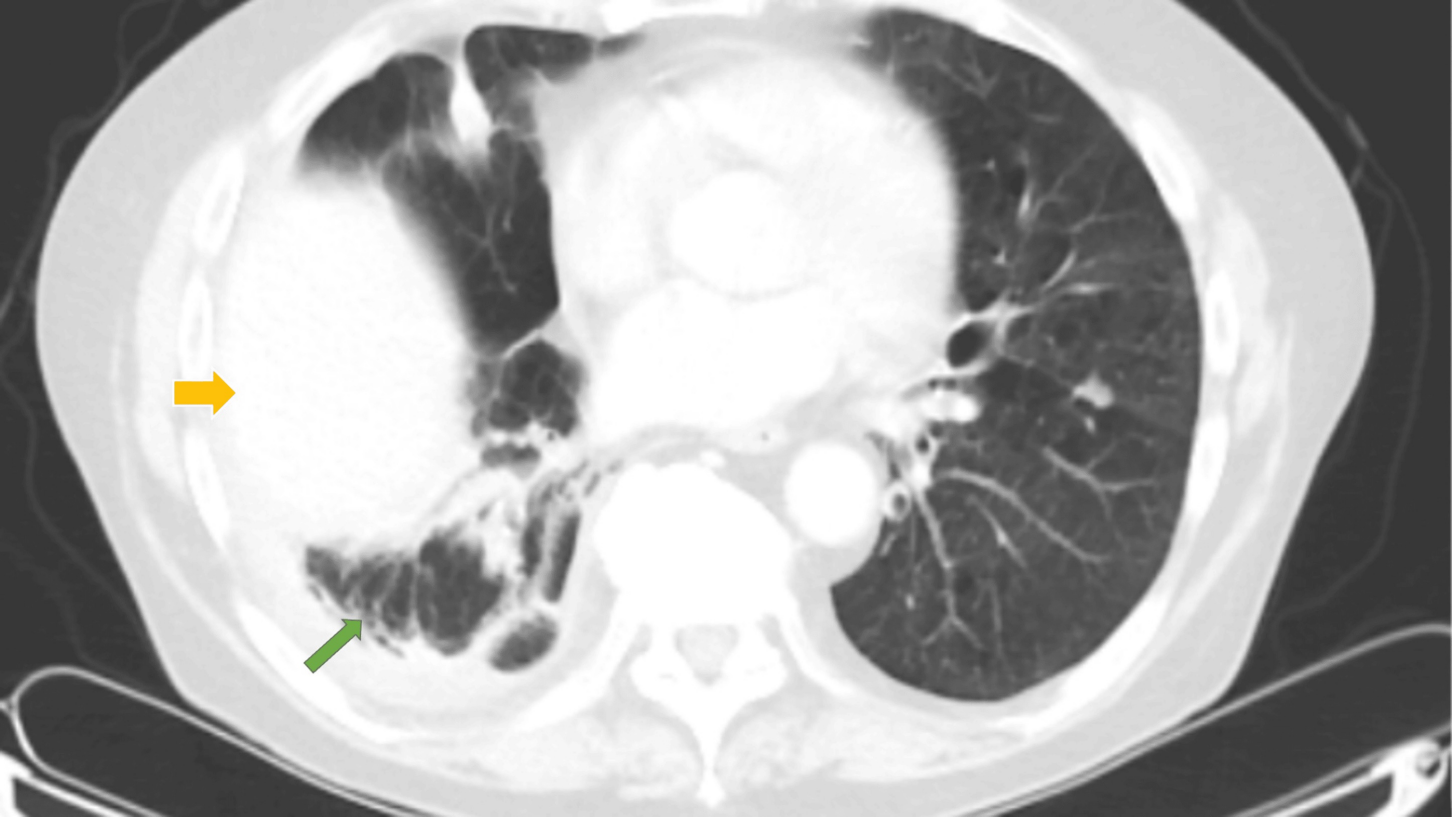
Follow-up CT of the thorax with contrast (25th June, 2025) showed interval improvement in right-sided empyema (yellow arrow), with a small residual loculated pleural collection and associated pleural thickening (green arrow).

At outpatient follow-up, he remained clinically well with improved exercise tolerance. Follow-up CT thorax scans on 25 June (Figure 4) and 24 July 2025 demonstrated interval resolution of the empyema cavity with residual bronchoceles and lingular cystic bronchiectasis, together with minor pleural thickening. An incidental multinodular goitre was also noted. Thoracic ultrasound in July confirmed a small, stable residual collection not requiring further drainage (Table 1).

**Table 2:** Summary of key laboratory investigations

Parameter	Result	Reference Range	Interpretation
White cell count (×10⁹/L)	40.3 (84% neutrophils)	3.6 – 9.2	Marked neutrophilic leukocytosis
C-reactive protein (mg/L)	187 (peak 392)	0 – 1.5	Significantly elevated inflammatory marker
Arterial pH	7.23	7.35 – 7.45	Acidemia
pCO₂ (kPa)	8.2	4.6 – 6.4	Respiratory acidosis
pO₂ (kPa)	10.9	11 – 14.4	Mild hypoxaemia
Pleural fluid pH	< 7.2	7.60–7.64	Acidotic, consistent with empyema

He was subsequently reviewed in the ENT clinic, where vocal-cord leukoplakia was again noted. Further diagnostic laryngoscopy and biopsy were recommended to rule out malignancy, but the patient declined due to distress from his previous bronchoscopy experience.

## Discussion

Empyema as a post-bronchoscopy complication is extremely rare. The presumed mechanism involves bacterial translocation from colonised airways into damaged parenchyma or pleura following instrumentation [2, 3]. During diagnostic flexible bronchoscopy, the instrument allows direct visualisation from the upper airways down to the segmental and subsegmental bronchi but does not reach the true peripheral lung. For bronchoalveolar lavage, the bronchoscope is typically wedged in a segmental or subsegmental (third- to fourth-order) bronchus, allowing saline to reach the distal bronchoalveolar units for sampling [5, 6]. Although the procedure itself does not extend to the lung periphery, lavage and suction manoeuvres can still translocate bacteria from colonised proximal airways into more distal, structurally abnormal regions. Patients with structural lung disease are particularly predisposed because of pre-existing colonisation and impaired clearance [4].

This patient had chronic bronchiectasis with prior colonisation by *S. aureus *and *P. septica*, organisms that may colonise structurally abnormal airways but only rarely cause invasive infection.

The delayed presentation (10 days after bronchoscopy) and the change in microbial profile from BAL to pleural fluid (*E. coli*) suggest secondary infection of the pleural space rather than direct inoculation during bronchoscopy.

An intriguing aspect of this case was the discrepancy between the airway and pleural isolates. BAL isolated *S. aureus* and *P. septica*, whereas the pleural aspirate grew only *E. coli*. Such discordance is well recognised in pleural infection [2]. The British Thoracic Society Pleural Disease Guideline emphasises that sputum and airway samples are unreliable for guiding therapy in empyema, as pleural-fluid microbiology often differs [2]. Similarly, in the U.K., a controlled trial of intrapleural streptokinase for pleural infection, only 58% of pleural-fluid samples were culture-positive, and many showed anaerobic or polymicrobial growth distinct from airway flora [7].

Pleural infection is frequently polymicrobial; however, when typical pathogens such as *S. aureus* or *Streptococcus pneumoniae* are isolated, the infection tends to be monomicrobial [8]. These findings emphasise that definitive antimicrobial management should always be guided by pleural-fluid cultures while considering potential additional pathogens.

The isolation of *P. septica *is noteworthy. It is an environmental Gram-negative bacillus rarely implicated in pulmonary infections but reported in immunocompromised or structurally abnormal lungs [3]. Empyema caused by *E. coli* is uncommon but recognised, particularly in polymicrobial infections or in association with aspiration events [9]. This case highlights how colonisation, airway instrumentation, and altered host defences can interact to produce unexpected infectious sequelae.

## Conclusions

Post-bronchoscopy empyema is an uncommon but serious complication, particularly in patients with structural lung disease. This case underscores the importance of early recognition of post-procedure infection, the limitations of airway samples in predicting pleural microbiology, and the need for tailored antibiotic therapy based on pleural-fluid culture. It also highlights the potential role of rare organisms such as *P. septica* in post-procedural infections. Vigilant follow-up and serial imaging are essential to monitor resolution and functional recovery.
